# Author’s reply: Regarding the editorial by Penttinen and Friede

**DOI:** 10.2807/1560-7917.ES.2016.21.40.30367

**Published:** 2016-10-06

**Authors:** Pasi M Penttinen, Martin H Friede

**Affiliations:** 1 European Centre for Disease Prevention and Control (ECDC), Stockholm, Sweden; 2 Initiative for Vaccine Research (IVR), World Health Organization (WHO), Geneva, Switzerland

**Keywords:** Keywords: influenza, vaccines and immunisation, vaccine effectiveness

**To the editor:** We thank Fry et al. for their interest in our editorial, in which we sought to lay out the evolving evidence surrounding the effectiveness of live attenuated influenza vaccine (LAIV) in preventing infections with influenza A(H1N1)pdm09 and the potential consequences of a perceived or true lack of effectiveness for childhood immunisation programmes and pandemic preparedness [1]. We appreciate that they meticulously highlight several errors in the Table and provided more accurate data which was not available to us through the original presentations from the United States (US) Advisory Committee on Immunization Practices (ACIP) [2]. All errors were corrected in the original text on 29 September and the more accurate sample sizes and confidence intervals were incorporated as suggested. In our view, these amendments do not change our conclusions. We look forward to the peer-reviewed publication of vaccine effectiveness (VE) studies done in the US as important contributions to the evidence base.

Fry et al. note that the VE estimate for Finland referred to all type A influenza, not specifically to influenza A(H1N1)pdm09. However, it is important to note that only 7% of subtyped influenza viruses during the 2015/16 season from Finland were influenza A(H3N2), and almost all of those came from adult patients [3]. Therefore, we assume that the VE estimate against influenza A in Finland accurately reflects the VE against influenza A(H1N1)pdm09 in children.

We note that Fry et al. agree with our final conclusion in that ‘*It is critical to understand why LAIV did not work as expected against the 2009 pandemic virus in the multivalent formulations*’ [4]. We suggest that the international scientific collaboration already established between the involved public health agencies under the coordination of the World Health Organization continues at an intensive pace in order to ensure that the scientific community, the public health community, policymakers and manufacturers resolve this critical question.
